# Numerical Analysis of the Impact Resistance of Composite A-Shaped Sandwich Structures

**DOI:** 10.3390/ma16145031

**Published:** 2023-07-16

**Authors:** Xuetao Gu, Jiawen Li, Ji Huang, Yaoliang Ao, Bingxiong Zhao

**Affiliations:** 1 Naval Architecture and Shipping College, Guangdong Ocean University, Zhanjiang 524088, China; ilovemamba@stu.gdou.edu.cn (X.G.); jiawen-li@gdou.edu.cn (J.L.); bxzhao@gdou.edu.cn (B.Z.); 2 Technical Research Center for Ship Intelligence and Safety Engineering of Guangdong Province, Guangdong Ocean University, Zhanjiang 524088, China; 3 China Classification Society Guangzhou Branch (CCS), Guangzhou 510235, China

**Keywords:** carbon fiber composites, A-shaped core sandwich structure, ballistic limit velocity, failure mode

## Abstract

This paper focuses on the finite element analysis simulation of the impact properties of composite sandwich structures made of carbon fiber-reinforced polymer lamina. In the existing studies, the composite sandwich structures with A-shaped cores have superior mechanical properties under quasi-static plane compression loads compared to W-shaped, Y-shaped, and X-shaped cores. However, there is limited research on the impact resistance of this structure. This paper studied the resistance of a composite A-shaped core structure to ballistic impact. Using ABAQUS/explicit finite element analysis software, ballistic impact tests for the composite A-shaped core structure were simulated based on the Hashin and Yeh failure criteria with a progressive damage model introduced in the user-defined subroutine VUMAT. First, the composite Y-shaped core sandwich structure was verified via experiments and simulations to determine the accuracy of the method, and then the composite A-shaped sandwich structure was subjected to a series of ballistic impact simulations. With varied impact velocity, the damage to the front and rear face sheet and cores via ballistic loads was simulated to illustrate the overall dynamic response process of the sandwich structure. Subsequently, a curve was fitted using a ballistic limit velocity equation, which was used as the criterion to evaluate the impact resistance of the composite A-shaped core structure. The results showed that, under the same relative density and the same number of component layers, the ballistic limit velocity of the composite A-shaped core sandwich structure was bigger than the composite Y-shaped core sandwich structure. The composite A-shaped core structure had 12.23% higher ballistic limit velocity than the composite Y-shaped core, indicating the impact resistance capabilities of the A-shaped core structure. In addition, the impact location’s effect on the impact response was investigated.

## 1. Introduction

Naval vessels will be attacked by air and underwater weapons when fighting on the surface. Strong impacts will cause serious damage to the hull and affect the vitality and combat effectiveness of the ship. Increasing the thickness or number of layers of a steel plate is not an optimal solution in the design and construction of modern naval vessel protection. As a result, new materials or structures must be incorporated into the design of the lightweight construction of naval vessels to improve their resistance to damage. In the new protection structure of naval vessels, various sandwich structures have been developed, including corrugated cores [1,2,3], honeycomb cores [4,5,6,7], and truss cores [8,9,10,11].

Qi et al. [12] designed and tested a tapered lattice truss core sandwich structure for out-of-plane and in-plane compression and shear to measure the stiffness and strength properties of the structure. The results showed that the out-of-plane compressive strength was determined by the buckling of the pillar. Guin et al. [13] investigated the effect of core shear joint width in honeycomb sandwich structures using tests and post-failure analysis. The results showed that the integrity of the face sheet-to-core interface degrades as the width of the core splice joint increases. Daliri et al. [14] investigated the effect of the wave number on the bending properties of bidirectional or regular corrugated core plates by proposing a bidirectional sinusoidal corrugated core plate. The results showed that the panel with *T* = 37.5 mm had the highest specific energy absorption.

Compared to honeycomb, truss, and foam cores, corrugated cores have superior bending and shear properties and energy absorption capacity [15]. As a result, different corrugated core configurations have been proposed, such as W-shaped, X-shaped, Y-shaped, V-shaped, and sinusoidal-shaped; they have been investigated for their suitability as lightweight cores. Furthermore, due to the carbon fiber composite sandwich structure’s chemical stability, corrosion resistance, and lightweight properties [16], it has been widely used in the marine, aerospace, and automotive sectors. Zhang [17] investigated the impact resistance of corrugated sandwich structures and found that when ultra-high molecular weight polyethylene (UHMWPE) fibers were used as the front panel, the structures had high impact resistance. When the composite material is used as the back panel, although the residual velocity is lower than that of metallic aluminum, the impact force is higher than that of metallic aluminum and the damage form of the target panel is more severe. Therefore, the impact resistance of the composite front panel/metal aluminum core/metal aluminum back panel sandwich structure is better than that of the all-metal aluminum corrugated sandwich structure. Zhao et al. [18] developed a new glass fiber-reinforced double-corrugated sandwich structure; they analyzed and discussed its damage mechanism and situation under low-velocity impact. The results showed that this structure exhibited a significant increase in the maximum impact load and energy absorption before structural failure with only a small increase in weight and a reduction in the indentation at low-velocity impacts. He et al. [3] investigated the effects of impact energy, impact site, and panel material type on the impact response and resulting damage state of the carbon fiber X-shaped core sandwich structure using experimental and numerical methods. The results showed that the composite sandwich structures displayed a higher impact energy capacity than aluminum sandwich structures. Yu et al. [19] studied the impact resistance of the carbon fiber-reinforced composite Y-shaped core sandwich structure by fitting the test data to obtain the ballistic limiting velocity through a series of impact tests; they discussed and analyzed the damage to the sandwich structure under impact. They also explored the effects of different impact locations on the impact response. The results showed that the Y-shaped core sandwich structure had greater ballistic limiting velocity compared to the carbon fiber composite laminate. The ballistic limit velocity of the composite sandwich structure increased by 15.52% compared to that of the laminate. In addition, Ren et al. [15] compared the mechanical properties of four different types of cores (W-shaped, Y-shaped, X-shaped, and A-shaped) under planar compressive loading using a numerical evaluation model validated by W-shaped cores and two parameter indicators, energy absorption (EA) and energy absorption efficiency (EAE). The results showed that A-shaped cores had higher EA and EAE than the other cores.

The above-mentioned literature suggests that the composite sandwich structure with A-shaped cores has superior mechanical properties under quasi-static plane compression loads compared to W-shaped, Y-shaped, and X-shaped. However, there is limited research on the impact resistance of this structure. To fill the gap in our understanding of the dynamic mechanical properties of A-shaped core sandwich structures, further research is needed to investigate the impact resistance and failure mode of this composite sandwich structure under ballistic impact load.

In this paper, a series of ballistic impact simulations were carried out on the composite A-shaped core sandwich structure using ABAQUS finite element analysis (FEA) software to analyze the damage to the front and rear panels and cores by ballistic loads with different impact velocities and to understand the overall dynamic response process of the core structures. The limit ballistic velocity was obtained by fitting the processed data and comparing them with the composite Y-shaped core sandwich structure. Finally, the impact location was also investigated to determine its effect on the impact response.

## 2. Theoretical Background

Based on the generic 3D failure criterion “Hashin” [20], which has good predictive accuracy for failure damage models, and the “Yeh” delamination criteria [21,22,23], a progressive failure model was developed in this paper. Both criteria are incorporated in the VUMAT user subroutine and ABAQUS/explicit calculation. The 3D Hashin failure criteria can account for the anisotropic nature of composite sandwich structures, among other characteristics; the criteria were selected to assess matrix failure and fiber fracture. In addition, the Yeh delamination criteria were used in the model to assess delamination failure. The strain-based Hashin failure criteria are as follows:

We define the fiber damage level caused by fiber tension $R_{\mathrm{ft}}$ as follows:

(1) $$\begin{array}{cc} R_{\mathrm{ft}}^{2} & ={\left(\frac{\varepsilon_{11}}{X_{T}^{\varepsilon }}\right)}^{2}+{\left(\frac{\varepsilon_{12}}{S_{12}^{\varepsilon }}\right)}^{2}+{\left(\frac{\varepsilon_{13}}{S_{13}^{\varepsilon }}\right)}^{2},\varepsilon_{11}\geq 0. \end{array}$$

The degree of fiber damage caused by fiber compression $R_{\mathrm{fc}}$ is defined as follows:

(2) $$\begin{array}{cc} R_{\mathrm{fc}}^{2} & ={\left(\frac{\varepsilon_{11}}{X_{C}^{\varepsilon }}\right)}^{2},\varepsilon_{11}<0. \end{array}$$

We define the matrix damage level due to matrix tensile $R_{\mathrm{mt}}$ as follows:

(3) $$\begin{array}{cc} R_{\mathrm{mt}}^{2}= & {\left(\frac{\varepsilon_{22}+\varepsilon_{33}}{Y_{T}^{\varepsilon }}\right)}^{2}+\left(\frac{1}{{\left(S_{23}^{\varepsilon }\right)}^{2}}\right)\left(\varepsilon_{23}^{2}-\frac{E_{22}E_{33}}{G_{23}^{2}}\varepsilon_{22}\varepsilon_{33}\right)+\\ \; & {\left(\frac{\varepsilon_{12}}{S_{12}^{\varepsilon }}\right)}^{2}+{\left(\frac{\varepsilon_{13}}{S_{13}^{\varepsilon }}\right)}^{2},\varepsilon_{22}+\varepsilon_{33}\geq 0. \end{array}$$

The matrix damage level, due to matrix compression $R_{\mathrm{mc}}$ is defined as follows:

(4) $$\begin{array}{cc} R_{\mathrm{mc}}^{2}= & \left(\frac{\varepsilon_{22}+\varepsilon_{33}}{Y_{C}^{\varepsilon }}\right)\left[{\left(\frac{E_{22}Y_{C}^{\varepsilon }}{2G_{12}S_{12}^{\varepsilon }}\right)}^{2}-1\right]+{\left(\frac{E_{22}\varepsilon_{22}+E_{33}\varepsilon_{33}}{2G_{12}S_{12}^{\varepsilon }}\right)}^{2}+\\ \; & {\left(\frac{\varepsilon_{12}}{S_{12}^{\varepsilon }}\right)}^{2}+{\left(\frac{\varepsilon_{13}}{S_{13}^{\varepsilon }}\right)}^{2}+\left(\frac{1}{{\left(S_{13}^{\varepsilon }\right)}^{2}}\right)\left(\varepsilon_{23}^{2}-\frac{E_{22}E_{33}}{G_{23}^{2}}\varepsilon_{22}\varepsilon_{33}\right),\varepsilon_{22}+\varepsilon_{33}<0. \end{array}$$

The compression effect and the extent of delamination failure $R_{\mathrm{ld}}$ are defined as follows:

(5) $$\begin{array}{cc} R_{\mathrm{ld}}^{2} & ={\left(\frac{\varepsilon_{33}}{Z_{T}^{\varepsilon }}\right)}^{2}+{\left(\frac{\varepsilon_{13}}{S_{13}^{\varepsilon }}\right)}^{2}+{\left(\frac{\varepsilon_{23}}{S_{23}^{\varepsilon }}\right)}^{2},\varepsilon_{33}\geq 0. \end{array}$$

where $\varepsilon_{11}$, $\varepsilon_{22}$, and $\varepsilon_{33}$ represent the strain along and perpendicular to the fiber direction under tensile or ballast loadings. $\varepsilon_{12}$, $\varepsilon_{13}$, and $\varepsilon_{23}$ represent the strain in-plane and out-of-plane. $X_{T}^{\epsilon }$, $X_{C}^{\epsilon }$, $Y_{T}^{\epsilon }$, and $Y_{C}^{\epsilon }$ represent the ultimate strains along and perpendicular to the fiber direction under tensile or ballast loadings, respectively. $S_{12}^{\epsilon }$, $S_{13}^{\epsilon }$, and $S_{23}^{\epsilon }$ represent the ultimate shear strain in-plane and out-of-plane, respectively, whereas $Z_{T}^{\epsilon }$ represents the ultimate tensile strain in the thickness direction. These strain parameters can be expressed as follows:

(6) $$\begin{array}{cc} \; & X_{T}^{\varepsilon }=X_{T}/E_{11},X_{C}^{\varepsilon }=X_{C}/E_{11}\;,\\ \; & Y_{T}^{\varepsilon }=Y_{T}/E_{22},Y_{C}^{\varepsilon }=Y_{C}/E_{22},Z_{T}^{\varepsilon }=Z_{T}/E_{33}\;,\\ \; & S_{12}^{\varepsilon }=S_{12}/G_{12},S_{13}^{\varepsilon }=S_{13}/G_{13},S_{23}^{\varepsilon }=S_{23}/G_{23}\;, \end{array}$$

$X_{T}$ and $X_{C}$ represent strength along and perpendicular to the fiber direction under tensile or ballast loadings, respectively. $Y_{T}$ and $Y_{C}$ represent in-plane and out-of-plane strength, respectively. $Z_{T}$ represents the strength in the thickness direction. $E_{11}$, $E_{22}$, and $E_{33}$ represent the modulus of elasticity along and perpendicular to the fiber direction under tensile or ballast loadings, respectively. $G_{12}$, $G_{13}$, and $G_{23}$ represent the shear modulus in-plane and out-of-plane, respectively. The damage initiation of the material point can be judged based on the state of $R_{i},\left(i=ft,fc,mt,mc,ld\right)$.

According to classical laminate theory, when the relationship $R_{i}\geq 1\left(i=ft,fc,mt,mc,ld\right)$ is satisfied, the corresponding failure damage occurs at the material point. The damage initiation of the material point will occur when any one of the five failure factors is satisfied, i.e., $R_{i}\geq 1\left(i=ft,fc,mt,mc,ld\right)$, but will not occur when all five failure factors are satisfied, i.e., $R_{i}<1\left(i=ft,fc,mt,mc,ld\right)$. However, this failure is not complete but occurs as a simultaneous degradation of the material stiffness matrix. In this numerical simulation, the degree of failure of the material point is reflected by the stiffness of the material point decreases. The damage variable parameters $d_{i},\left(i=ft,fc,mt,mc,ld\right)$, which are related to the failure factor $R_{i}$, are, therefore, set to indicate the degree of failure.

(7) $$d_{i}=1-\frac{1}{R_{i}^{n}}\left(R_{i}\geq 1,n\geq 1;i=ft,fc,mt,mc,ld\right).$$

To prevent the damage state from being restored, the historical damage parameter $d_{i}^{t}$ at time of *t* is used as follows:

(8) $$d_{i}^{t}=\max\left(d_{i}^{t},0\right),\left(\tau \leq t;i=ft,fc,mt,mc,ld\right).$$

During the iterations of the finite element software calculations, the material points begin to fail from damage when certain strain levels are reached at the cell integration points, as determined by the Hashin failure criterion and the Yeh delamination failure criterion. As a result, the material stiffness matrix begins to decrease. A set of degradation parameters is, therefore, introduced to establish the relationship between the progressive damage model and the degradation of the material stiffness matrix, as shown in Equation (9).

(9) $$\begin{array}{cc} {\left\{\varepsilon_{11}\;\varepsilon_{22}\;\varepsilon_{33}\;\gamma_{12}\;\gamma_{23}\;\gamma_{13}\right\}}^{T}= & \; \end{array}\;\left\{\begin{array}{cccccc} \frac{1}{E_{11}\left(1-w_{1}\right)}\; & -\frac{v_{12}}{E_{22}}\; & -\frac{v_{13}}{E_{33}} & 0 & 0 & 0\\ -\frac{v_{12}}{E_{22}} & \frac{1}{E_{22}\left(1-w_{2}\right)} & -\frac{v_{23}}{E_{22}} & 0 & 0 & 0\\ -\frac{v_{13}}{E_{33}} & -\frac{v_{23}}{E_{22}} & \frac{1}{E_{33}\left(1-w_{3}\right)} & 0 & 0 & 0\\ 0 & 0 & 0 & \frac{1}{G_{12}\left(1-w_{4}\right)} & 0 & 0\\ 0 & 0 & 0 & 0 & \frac{1}{G_{23}\left(1-w_{5}\right)} & 0\\ 0 & 0 & 0 & 0 & 0 & \frac{1}{G_{13}\left(1-w_{6}\right)} \end{array}\right\}\left\{\begin{array}{c} \sigma_{11}\\ \sigma_{22}\\ \sigma_{33}\\ \sigma_{12}\\ \sigma_{23}\\ \sigma_{13} \end{array}\right\},$$

where $w_{1}$ is the max between 0.0 and $d_{f}$; $w_{2}$ is the max among 0.0, $d_{f}$, and $d_{m}$; $w_{3}$ is the max among 0.0, $d_{f}$, and $d_{d}$. $w_{1}$ equals $w_{2}$; $w_{5}$ and $w_{6}$ are equal to $w_{3}$. $d_{f}$ is the max among 0.0, $d_{ft}$, and $d_{fc}$; $d_{m}$ is the max among 0.0, $d_{mt}$, and $d_{mc}$; $d_{d}$ is the max between 0.0 and $d_{ld}$.

According to the degraded stiffness matrix, when the damage parameter degrades to zero, the material point is considered to have failed and no longer contributes to the structural stiffness. As a result, this material point will be removed from the finite element model.

## 3. Numerical Model Building

**Figure 1:**
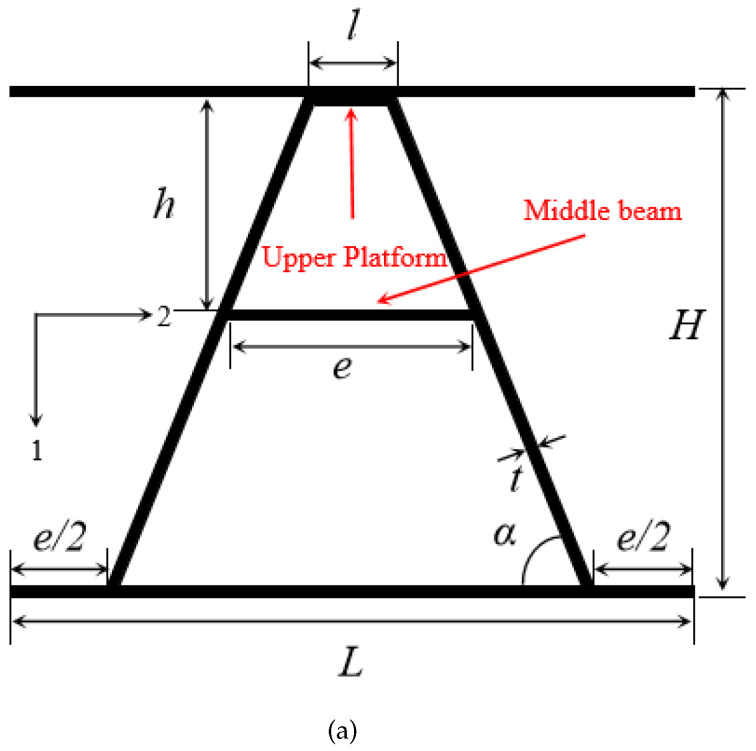

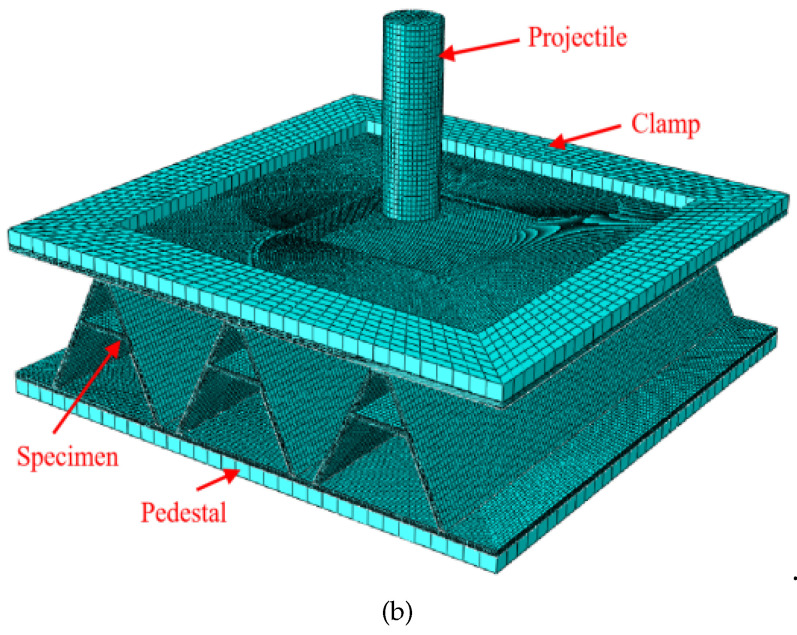
Composite A-shaped sandwich structure model. (**a**) A unit cell schematic of the composite sandwich structure with A-shaped cores. (**b**) Numerical simulation model of the composite sandwich structure with A-shaped.

In order to avoid the influences of different relative densities on the numerical simulation results of the impact resistance of the composite sandwich structure, we constructed a numerical model of the composite A-type core structure so that the relative density of the core structure matches that of the existing experimental Y-shaped core structure [19]. Since the structure was prepared using a hot-press molding technique via an integrated molding process, it was modeled in ABAQUS using an integrated model. The unit cell configuration of the composite sandwich structure with A-shaped cores is presented in Figure 1a. To clarify the characteristics of the A-shaped core, a single cell of the carbon fiber A-shaped core sandwich structure was developed. A right-angle coordinate system in the plane is established, where the cross-section of the single cell is located such that direction 1 is oriented vertically downwards in the plane and direction 2 is oriented to the right in the coordinate system, with directions 1 and 2 perpendicular to each other in the plane. The A-shaped core consists mainly of a triangular structure in the upper part and lower parts with inclined legs on both sides. This structure is symmetrical from side to side as shown in Figure 1b. The upper half of the structure connecting the left and right sides consists of the upper platform and the middle beam, with a width of *l* and *e*, respectively. The middle beam is *h* from the upper panel. The parameters α, *t*, and *H* represent the angle between the inclined leg and the horizontal on both sides, the thickness of the member, and the overall height of the composite sandwich construction with A-shaped cores, respectively.

In addition, the specific values of the aforementioned structural geometrical parameters are presented in Table 1. Based on the geometrical properties of the composite A-shaped core sandwich structure and the calculation formula of relative density in existing studies [19], the relative densities of the monoliths were calculated using the following equations.

(10) $$\begin{array}{cc} \; & \rho =\frac{2H\sin^{-1}\alpha +e+l}{HL}t\;,\\ \; & L=2\left(H-h\right)\cot\alpha +2e+2t\;,\\ \; & e=2h\cot\alpha +l. \end{array}$$

The constituent members of the A-shaped cores and face sheet were composed of 12 layers of unidirectional carbon/epoxy pre-pregs stacked together in the following sequence: ${\left[0^{\circ }/90^{\circ }/0^{\circ }/90^{\circ }/0^{\circ }/90^{\circ }\right]}_{s}$. The thickness of the panels and core members is 1.2 mm. The material and strength parameters for the panels and core layers are shown in Table 2. In addition, the relative density of the composite A-shaped sandwich structure was calculated to be 9.93% according to Equation (11), which indicates that the relative density and layup order of the composite A-shaped sandwich structure in this study are the same as the existing experimental Y-shaped core structure [19].

The core layer is often bonded to the core layer using the epoxy resin adhesive. Therefore, in the ABAQUS simulation software, for the finite element simulation model of this A-shaped core composite sandwich structure, the interaction property between the face sheets and cores was the ‘tie constraint’, which was used to simulate the true connected relation between the cores and face sheets. During bullet impact, the clamps on the outside of the panel were constrained to prevent movement in all directions. General contacts were adopted on potential contact surfaces to avoid mesh penetration. The friction coefficient value of the tangential friction interaction was 0.3. In addition, the initial velocity of the projectile was set using the predefined velocity field.

The numerical model of the flush cylindrical cartridge, fixture, and composite A-shaped core sandwich structure is shown in Figure 1b. To define the mesh for the simulation model, the solid element C3D8R (8-node linear brick, reduced-integration element) was used to construct the top and bottom panels and cores of the specimen, as hexahedral cells produce more accurate stress results in the non-planar direction than shell cells [25]. The fixture and cartridge were modeled as steel with Young’s modulus $E_{S}^{steel}$ = 210 Gpa with Poisson’s ratio of 0.3 and density of ρ = 7.9 ×$10^{-9}$ t/mm${}^{3}$. In the finite element model, the mesh sizes of the clamps and pedestals, cartridge body, and sandwich structure were approximately 2.5 mm × 2.3 mm × 3 mm, 1 mm × 1 mm × 1 mm, and 1 mm × 1 mm × 0.1 mm, respectively. Because of the limitations of the impact test, the core had three single cells in the transverse direction with the panel surface measuring 120 mm × 120 mm and an effective impact area of the projectile of 90 mm × 90 mm. In order to reduce the computation amount and computation time, only the impact area grid was encrypted. According to the experimental environment of the test, a flat-headed cylindrical projectile was chosen. The projectile had a diameter of 12.67 mm, a height of 34.48 mm, and a weight of 34.33 g. The bullet had a diameter of 12.67 mm, a height of 34.48 mm, and a weight of 34.33 g.

## 4. Analysis of Results and Discussion

### 4.1. Ballistic Limit Velocity

The ballistic limit velocity was estimated by fitting a function between the initial incidence velocity and the residual velocity using the least squares method. The Lambert–Jonas equation [26,27] was utilized to obtain the ballistic limit velocity and evaluate the impact resistance performance of the sandwich structure. The Lambert–Jonas equation can be expressed as Equation (11):

(11) $$v_{r}=\{\begin{array}{c} 0,v_{i}\leq v_{bl}\;,\\ A{\left(v_{i}^{p}-v_{bl}^{p}\right)}^{1/p},v_{i}>v_{bl}\;, \end{array}$$

where $v_{bl}$, *A*, and *p* are the fitting parameters. The initial velocity $v_{i}$ and residual velocity $v_{r}$ are shown in Table 3. This expression had significance only when $v_{i}>v_{bl}$ and $v_{bl}$ were the ballistic limit velocities obtained by fitting the equation. If the projectile failed to penetrate the sandwich structure ($v_{i}\leq v_{bl}$), the residual velocity value of the projectile was defined as zero [28].

#### 4.1.1. Verification of the Ballistic Limit Velocity Model

**Figure 2:**
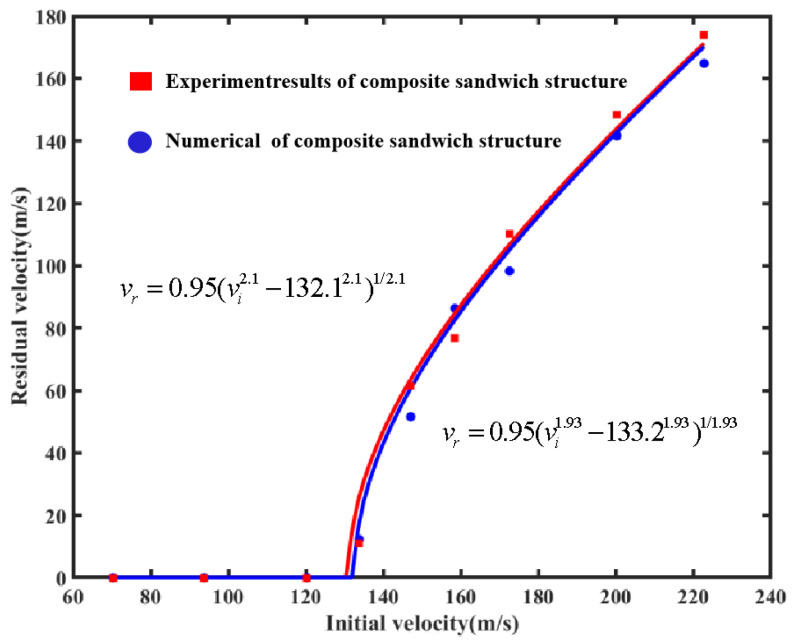
Residual velocity vs. impact initial velocity for the numerical and experiment results of the composite sandwich structure with Y-shaped cores.

The numerical analysis method for the simulation in this work was validated by comparing it with previous experiments [19]. As shown in Table 3, the comparison between the experimental residual velocity and the simulation revealed a maximum error and minimum error in the residual velocities of 18.99% and 4.80%, respectively. The fitted curves for the existing experiments [19] and the numerical simulations are shown in Figure 2. The ballistic limit velocity obtained by fitting the numerical simulation results was 132.1 m/s, with a relative error of 0.83%, compared to the experimental result of 133.2 m/s. The two results are considered to be in good agreement and reliable.

This study essentially yielded impact resistance results similar to previous studies on the failure mode of the Y-shaped core composite sandwich structure [19]. In addition, when the numerical simulation and experimental results were compared, the maximum error and minimum error of residual velocities were 18.99% and 4.80%, respectively. As the relative error between the two ballistic limit velocities was only 0.83%, the simulation results had a high degree of reliability. Due to the slight differences in the processing of the carbon fiber composite laminate and the differences in boundary conditions between the simulation and the experiment, although the initial velocity was the same, there were some differences between the residual velocity obtained via numerical simulation and the experimental results.

#### 4.1.2. Comparison of the Ballistic Limit Velocity of A-Shaped Sandwich Structures and Y-Shaped Sandwich Structures

A series of ballistic impact simulations were carried out on the carbon fiber A-shaped core composite sandwich structure to obtain the initial and residual velocities at different impact velocities. The initial residual velocity results for the Y-shaped and A-shaped core composite sandwich structures are shown in Table 4.

**Figure 3:**
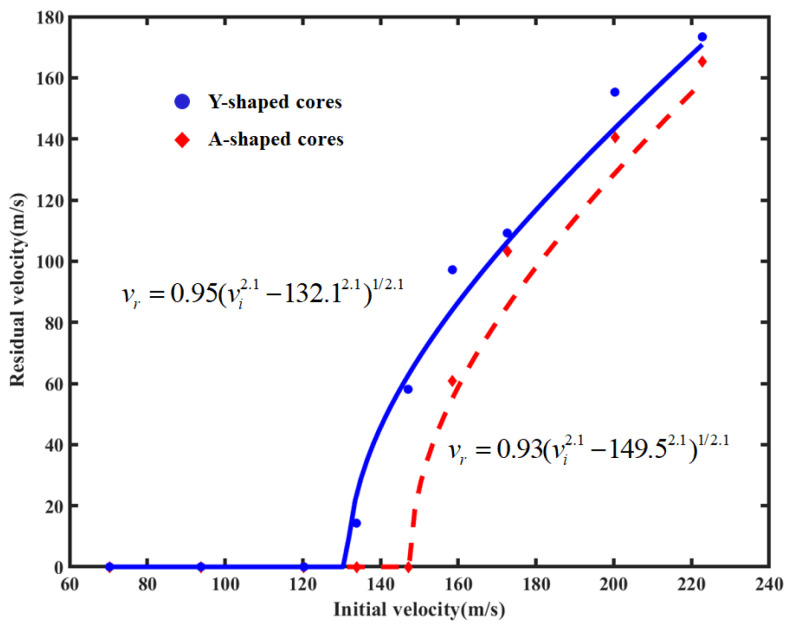
Residual velocity vs. impact initial velocity for two sandwich structures.

The data in Table 4 were fitted in the Lambert–Jonas equation using MATLAB to produce a plot of the initial residual velocity profiles for the composite sandwich structures with Y-shaped cores and A-shaped cores, as shown in Figure 3. The ballistic limit velocity of the A-shaped core composite sandwich structures was 149.5 m/s, which was 12.23% higher than 133.2 m/s for the Y-shaped core composite sandwich structures. This clearly implies that the A-shaped core composite sandwich structures have better impact resistance under impact loading.

### 4.2. Failure Mode

The numerical simulation showed that the composite A-shaped core sandwich structure had two structural damage modes during this ballistic impact, based on the initial kinetic energy of the projectile. The first mode involved incomplete penetration, where the front face sheet was penetrated and the core partially failed, but the bullet eventually rebounded from or remained within the core structure. The second mode was the complete penetration, where the bullet completely penetrated the rear face sheet, indicating that it had completely penetrated the entire core structure.

#### 4.2.1. Failure Mode Verification of Structures

**Figure 4:**
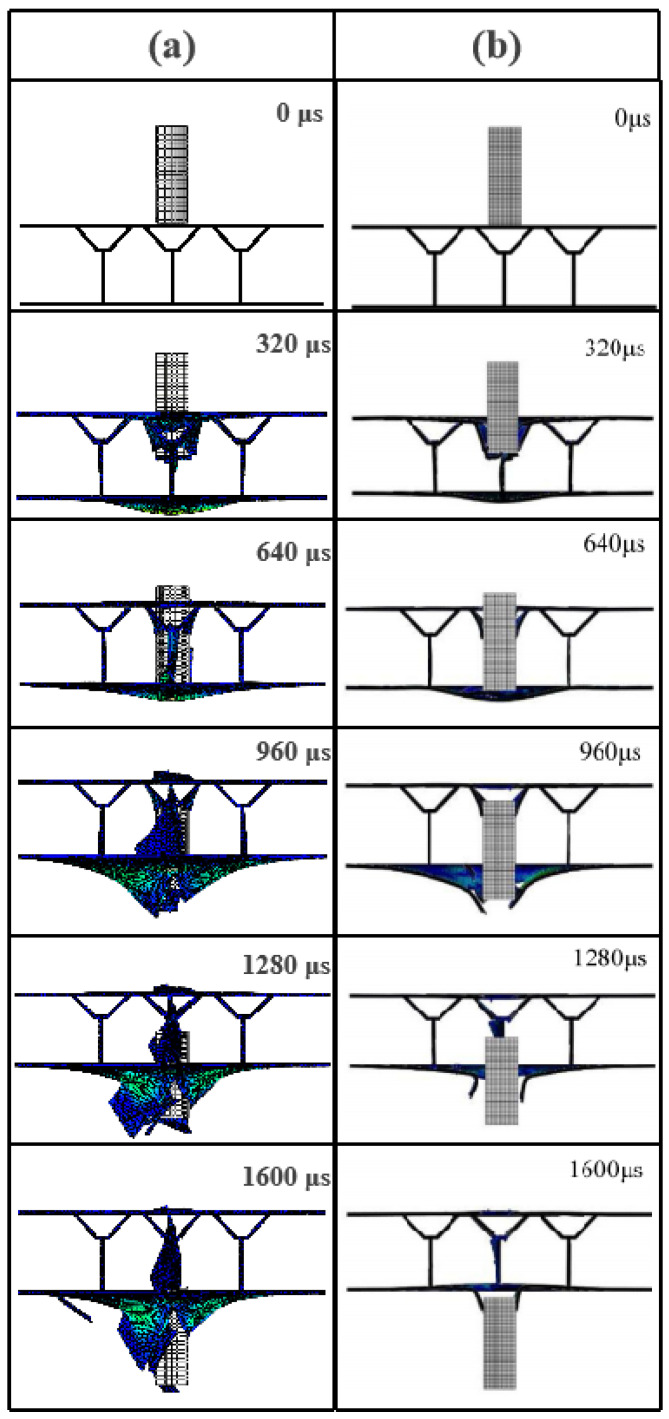
Comparative diagram of the impact response process of the Y-shaped composite sandwich structure at *v* = 133.8 m/s: (**a**) our numerical simulation results; (**b**) numerical simulation results of existing studies [19].

**Figure 5:**
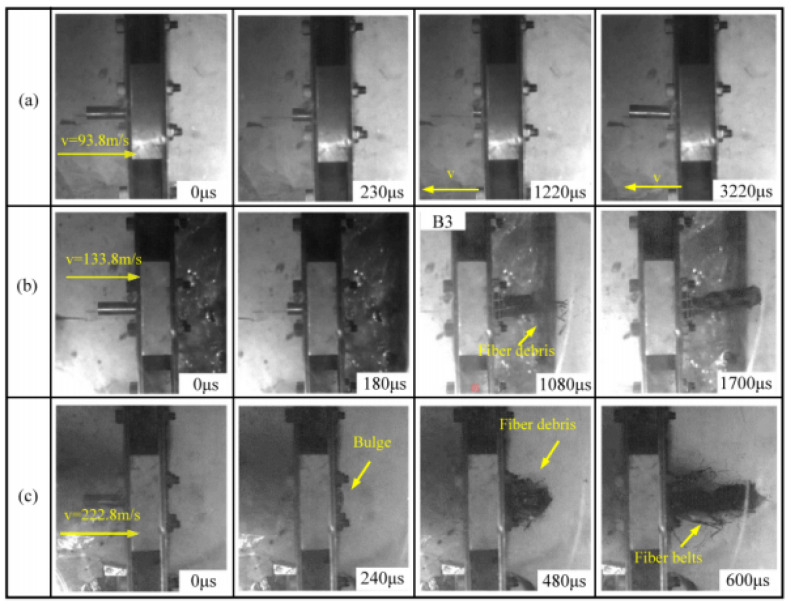
The impact process of the Y-shaped sandwich structure in the existing experiment [19]: (**a**) *v* = 93.8 m/s; (**b**) *v* = 133.8 m/s; (**c**) *v* = 222.8 m/s.

**Figure 6:**
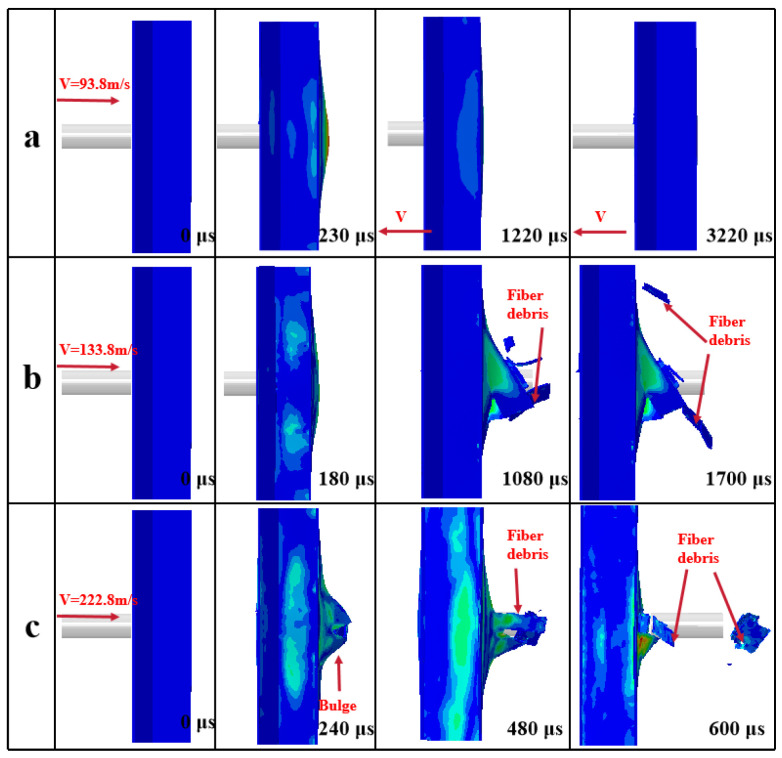
Simulation of the impact process of the Y-shaped sandwich structure: (**a**) *v* = 93.8 m/s; (**b**) *v* = 133.8 m/s; (**c**) *v* = 222.8 m/s.

The impact response of the Y-composite sandwich structure obtained by numerical simulation in this paper and existing at 133.8 m/s is shown Figure 4a,b, respectively. As can be seen in Figure 4a,b, the basic responses to the impact resistance processes of the two Y-shaped core composite sandwich structures were essentially the same from 0 to 640 μs. However, from 960 to 1600 μs, the impact resistance response processes were more intense and a plug was formed at the top of the projectile (Figure 4a). A comparison between the research method used in this paper and the impact processes of previous experiments [19] showed that, compared to the numerical simulation, the impact process was similar to experimental results at 93.8 m/s (Figure 5 and Figure 6) but more intense at 133.8 m/s and 222.8 m/s. However, in general, similar bulges and fiber debris were generated. Therefore, it can be considered that the failure mode of the Y-shaped sandwich structure simulated by us is basically the same as that of the experiment [19].

#### 4.2.2. Impact Damage Process for the Composite A-Shaped Core Sandwich Structures

**Figure 7:**
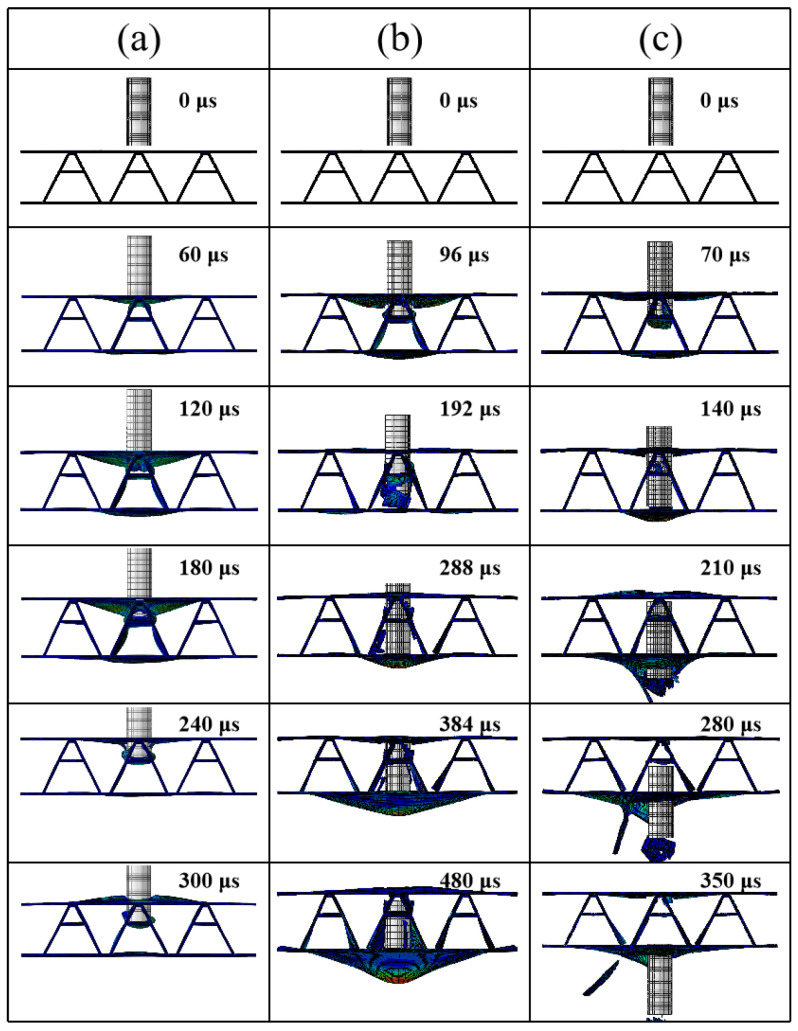
Damage destruction process of the A-shaped core composite sandwich structure under different impact velocities: (**a**) *v* = 93.8 m/s; (**b**) *v* = 133.8 m/s; (**c**) *v* = 222.8 m/s.

The damage process of this sandwich structure at both low near-ballistic limit velocity and high-impact velocity is shown in Figure 7. The damage process of the sandwich structure at an impact velocity of 93.8 m/s is shown in Figure 7a. Briefly, during the initial period (0–180 μs), the front face sheet underwent violent deformation and delamination failure on both sides of the inclined legs of the core. From 240 μs onward, the deformation of the structure diminished, and at 300 μs, the front face sheet experienced delamination failure and the bullet did not penetrate the middle beam. The front face sheet also showed delamination failure at 300 μs. The damage process of the sandwich structure at an impact velocity of 147.1 m/s is shown in Figure 7b. At 96 μs, the bullet penetrated the front face sheet and started to impact the middle beam. By 192 μs, it penetrated the middle beam and caused a fiber fracture, with little remaining kinetic energy. The bullet continued to impact the structure until 480 μs, when the rear face sheet was at its maximum deformation and the bullet could not penetrate the structure any further and began to rebound.

#### 4.2.3. Damage to the Face Sheet, Rear Sheet, and A-Shaped Core

**Figure 8:**
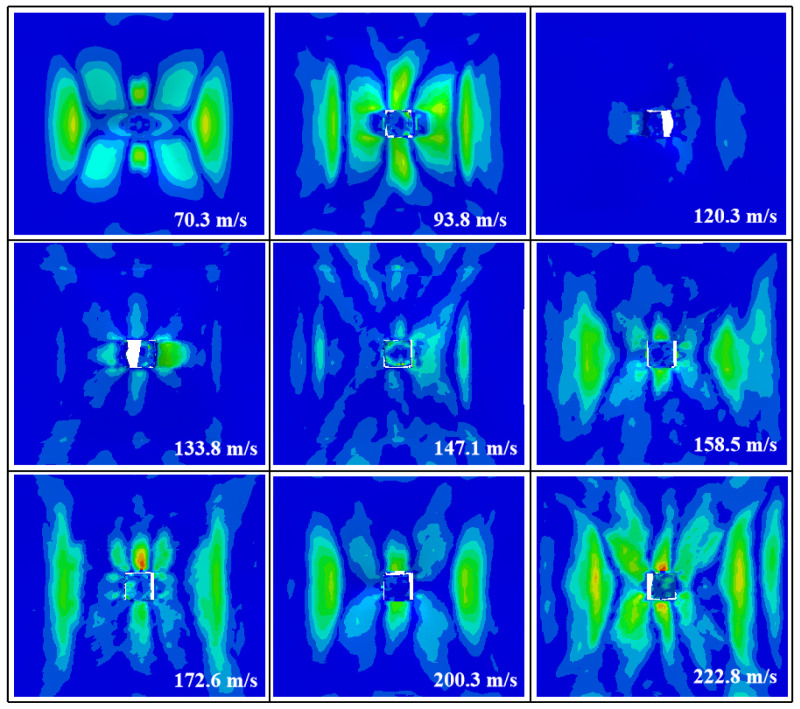
The failure modes of the front face sheet of the composite sandwich structure with A-shaped cores in the numerical simulation at different impact velocities.

A numerical simulation of the damage conditions to the front face sheet at different impact velocities is shown in Figure 8. The results show that the front face sheet was penetrated at all velocities other than 70.3 m/s. The perforations were square in shape and fiber fragments could be seen at the edges of the holes. The stress distribution on the front face sheet was more symmetrical at all velocities, except for 120.3 m/s.

**Figure 9:**
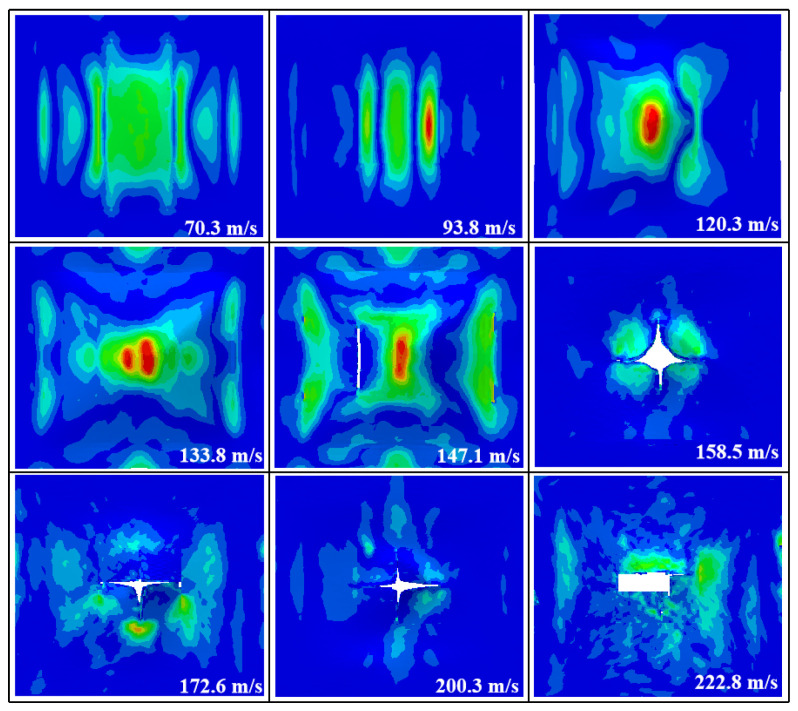
The failure modes of the rear face sheet of the composite sandwich structure with A-shaped cores in the numerical simulation at different impact velocities.

A numerical simulation of the damage conditions to the rear face sheet at different impact velocities is shown in Figure 9. The results show that at speeds of 70.3 m/s and 93.8 m/s, the damage to the rear face sheet was not obvious. However, at higher speeds of 120.3 m/s and 133.8 m/s, a bulge appeared on the rear face sheet. At 147.1 m/s, a crack developed on the left side of the rear face sheet, and at 158.5 m/s, the rear face sheet was penetrated and perforated in the shape of a “+”.

**Figure 10:**
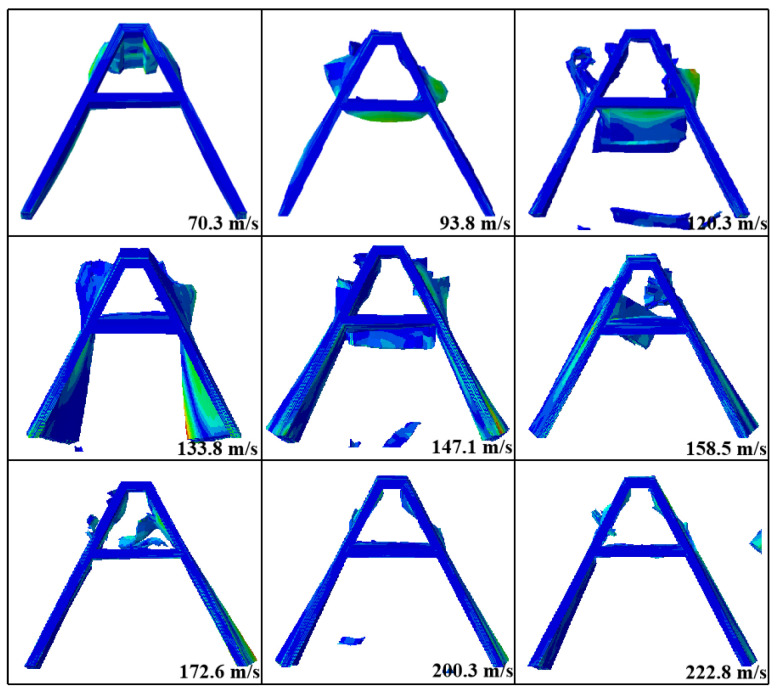
The failure modes of the back face sheet of the A-shaped cores in the numerical simulation at different impact velocities.

A numerical simulation of damage conditions to the A-shaped core at different impact velocities is shown in Figure 10. The results show that as the velocity increased, the damage to the core became more severe. At 70.3 m/s, the upper platform of the A-shaped core was depressed and fibers were withdrawn from the sides. At 93.8 m/s, the upper platform of the core was penetrated and the middle beam bulged downwards due to the continued impact of the bullet. At 120.3 m/s, the bullet penetrated the middle beam, causing it to fracture and delaminate the fibers. In addition, the diagonal side legs fractured from the upper platform but the core surface did not delaminate. At 133.8–172.6 m/s, delamination occurred at the two diagonal legs of the core, mainly due to the concentration of stress at the joints between the diagonal legs and the rear face sheet, and the large amount of fiber fragments produced at the middle beam. Delamination became evident at 200.3 m/s and 222.8 m/s in the lower parts of both inclined lateral legs but not in the upper parts.

It can be concluded that the upper platform, the upper part of the two inclined side legs, and the middle beam were crucial in resisting low-speed impacts, whereas the lower parts of the two inclined side legs played important roles in resisting high-speed impacts.

### 4.3. Numerical Simulation of the Impact Position

**Figure 11:**
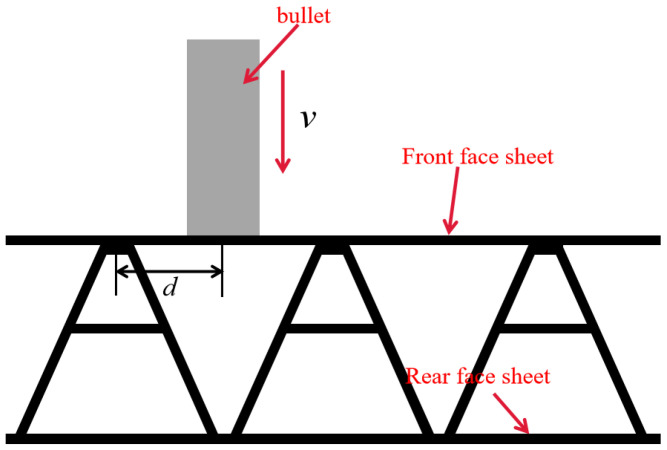
Schematic diagram of the impact position.

**Figure 12:**
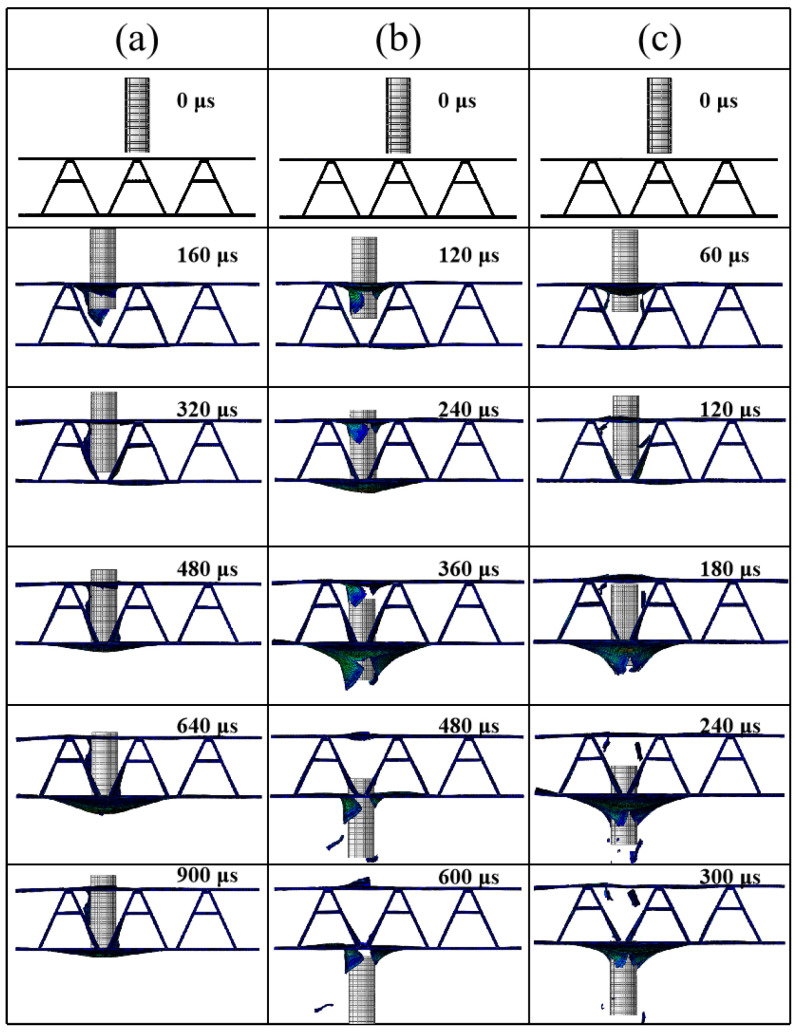
Numerical simulation results of the failure process of the A-shaped composite sandwich structure impacting the halfway point between cores at different impact velocities: (**a**) *v* = 93.8 m/s; (**b**) *v* = 147.1 m/s; (**c**) *v* = 222.8 m/s.

During the ballistic impact, the damage to the sandwich structure varied depending on the impact location. The position of the impact at the midpoint between the two A-shaped core types is shown in Figure 11. The distance from the impact position to the center line of the A-shaped core was 13.6 mm. The comparison of the effect of the impact position on the impact failure process of the composite sandwich structure showed that the initial impact velocities for the projectile were the same for both A-shaped core types when impacted at the halfway point and the center of the specimen (Figure 7). The impact process (at the midpoint of the two cores at different impact speeds) is shown in Figure 12. As can be seen in Figure 12a, at 160 μs, a bullet with an impact velocity of 93.8 m/s penetrated the rear face sheet and continued to impact it. At 640 μs, the rear face sheet developed a maximum bulge and the lower parts of the legs on both sides were damaged, resulting in the production of fiber fragments. At this point, the bullet began to rebound. As shown in Figure 12b, at 120 μs, the bullet with an impact velocity of 147.1 m/s penetrated the front face sheet, and at 360, the rear face sheet was also penetrated. As can be seen in Figure 12c, at 60 μs, the bullet with an impact velocity of 222.8 m/s penetrated the front face sheet and continued to impact it. At 120 μs, delamination occurred in the lower part of the legs on both sides, and at 240 μs, complete penetration of the core structure occurred. As can be seen in Figure 7, changing the impact position to the midpoint of the two cores decreased the impact resistance capabilities of the sandwich structure. Further, the crossbeam of the A-shaped core basically had no resistance role, and only a small part of the legs on both sides played a resistance role.

## 5. Conclusions

In this paper, we focused on the A-shaped core sandwich structure of carbon fiber composites. First, the experiments were simulated numerically using ABAQUS FEA software based on the existing impact experimental data for the Y-shaped core sandwich structure [19]. A comparison of errors in ballistic limit velocity and residual velocity and a comparison of the damage modes of the impact resistance process of the carbon fiber composite Y-shaped core sandwich structure between the numerical simulation and the experiment showed that numerical simulation results agreed well with the experimental results, supporting the accuracy of the numerical simulation method in this paper. Furthermore, the numerical simulation method was used to construct a numerical model of the carbon fiber composite A-shaped core structure and conduct a series of ballistic impact simulations. The results showed that the front face sheet was penetrated at 93.8 m/s, whereas the rear face sheet was penetrated at 158.5 m/s, reflecting the excellent impact resistance of the A-shaped core. In addition, the front face sheet perforation was rectangular, whereas the rear face sheet perforation was cross-shaped. The upper platform of the A-shaped core, the upper part of the inclined side leg, and the middle beam were crucial in resisting low-speed impacts, whereas the lower part of the inclined side leg played a more pronounced role in resisting high-speed impacts.

The ballistic limit velocity of the A-shaped core composite sandwich structures was 149.5 m/s, which was 12.23% higher than 133.2 m/s for the Y-shaped core composite sandwich structures. This clearly implied that the A-shaped core composite sandwich structures had better impact resistance under impact loading.

Finally, numerical simulations were carried out for different impact positions. The results showed that changing the impact position to the midpoint of the two cores decreased the impact resistance of the sandwich structure, and only a small part of the legs on both sides contributed to resistance, while the crossbeam of the A-shaped core played virtually no resistance role.

## Figures and Tables

**Table 1:** Geometric parameters of the unit cell of the A-shaped core.

Category	Symbol	Value
Upper platform (mm)	*l*	4.1
Mid-platform (mm)	*e*	10.4
Height of middle beam (mm)	*h*	9.0
Overall height of the sandwich structure (mm)	*H*	27.0
Thickness (mm)	*t*	1.2
Inclined angle of legs (${}^{\circ }$)	α	60

**Table 2:** Mechanical properties of the carbon fiber-reinforced polymer lamina [24].

Category	Symbol	Value
Longitudinal modulus (MPa)	$E_{11}$	100,000
Transverse modulus (MPa)	$E_{22}$	8000
Out-of-plane modulus (MPa)	$E_{33}$	8000
Poisson’s ratio	$V_{12},V_{13},V_{23}$	0.21, 0.21, 0.3
Shear modulus (MPa)	$G_{12},G_{13},G_{23}$	0.21, 0.21, 0.3
Shear strength (MPa)	$S_{12},S_{13},S_{23}$	104, 104, 86
Longitudinal tensile strength (MPa)	$X_{T}$	2100
Longitudinal compressive strength (MPa)	$X_{C}$	700
Transverse tensile strength (MPa)	$Y_{T}$	42
Transverse compressive strength (MPa)	$X_{C}$	160
Out-of-plane tensile strength (MPa)	$Z_{T}$	42
Out-of-plane compressive strength (MPa)	$Z_{C}$	1600
Density (kg/m${}^{3}$)	ρ	1500

**Table 3:** Residual velocity vs. impact initial velocity for the numerical and experiment results of the composite sandwich structure with Y-shaped cores.

Test Group	Experimental Results of Composite Sandwich Structure [19]		Numerical Results of Composite Sandwich Structure		Error (%)
	$v_{i}$ (m/s)	$v_{r}$ (m/s)	$v_{i}$ (m/s)	$v_{r}$ (m/s)	
1	70.3	–	70.3	–	-
2	93.8	–	93.8	–	-
3	120.3	–	120.3	–	-
4	133.8	12.3	133.8	10.9	11.38
5	147.1	51.6	147.1	61.4	18.99
6	158.5	86.3	158.5	76.7	11.12
7	172.6	98.3	172.6	110.1	12
8	200.3	141.6	200.3	148.4	4.8
9	222.8	164.8	222.8	174.3	5.76

**Table 4:** Initial residual velocity results for Y- and A-shaped core sandwich structures.

Test Group	Numerical Results of the Composite Y-Shaped Core Sandwich Structure		Numerical Results of the Composite A-Shaped Core Sandwich Structure	
	$v_{i}$ (m/s)	$v_{r}$ (m/s)	$v_{i}$ (m/s)	$v_{r}$ (m/s)
1	70.3	0	70.3	0
2	93.8	0	93.8	0
3	120.3	0	120.3	0
4	133.8	14.3	133.8	0
5	147.1	58.1	147.1	0
6	158.5	97.2	158.5	60.7
7	172.6	109.2	172.6	103.3
8	200.3	155.3	200.3	140.6
9	222.8	173.4	222.8	165.4

## Data Availability

Data will be provided upon request by the corresponding author via email.
